# Circulating Tumor DNA Combining with Imaging Analysis for Lesion Detection of Langerhans Cell Histiocytosis in Children

**DOI:** 10.3390/children11121449

**Published:** 2024-11-27

**Authors:** Siying Liu, Yongbing Zhu, Yu Chen, Yaqin Wang, Dedong Zhang, Jiasi Zhang, Yao Wang, Ai Zhang, Qun Hu, Aiguo Liu

**Affiliations:** Department of Pediatrics, Tongji Hospital, Tongji Medical College, Huazhong University of Science and Technology, Wuhan 430030, China

**Keywords:** Langerhans cell histiocytosis, liquid biopsy, children, cfBRAF^V600E^, imaging response

## Abstract

Background: The detection of mutations from circulating tumor DNA (ctDNA) represents a promising enrichment technique. In this retrospective study, the significance of ctDNA and imaging in Langerhans cell histiocytosis (LCH) monitoring was first examined, and the broader role of ctDNA in monitoring LCH was additionally explored. Methods: First, data visualization and survival analysis models were used to generalize the concordance between cfBRAF^V600E^ molecular response and radiographic response on clinical outcomes. Next, the molecular response of cfBRAF^V600E^ was observed from a dynamic perspective. A comparative analysis was then conducted between cfBRAF^V600E^ and ltBRAF^V600E^ status, examining their relationship to clinical manifestations and prognosis of LCH. Results: Eventually, 119 participants were enrolled in this trial between 2019 and 2023. Progression-free survival (PFS) was significantly shorter in patients with both radiologic and cfDNA molecular progression (17.67 versus 24.67 months, *p* < 0.05) compared to those without. A critical cfBRAF^V600E^ value of 0.03% has been determined for the first time. Both cfBRAF^V600E^ and ltBRAF^V600E^ mutations were associated with a higher proportion of children under 3 years of age, skin and spleen involvement, and a lower 3-year PFS rate. In contrast to ltBRAF^V600E^, cfBRAF^V600E^ was linked to a higher proportion of risk organ invasion LCH (52% vs. 27.9%, *p* < 0.05) and a better therapeutic response at the sixth week (24% vs. 4.7%, *p* < 0.05). Furthermore, in patients with risk organ invasion-LCH and multisystem-LCH subtypes, cfBRAF^V600E^ was associated with a significantly lower 3-year PFS. Conclusions: In summary, these findings enhanced and supplemented the implications of ctDNA and imaging analysis application in children with LCH.

## 1. Introduction

Langerhans cell histiocytosis (LCH) is the most prevalent form of childhood histiocytosis, characterized by CD207-positive histiocytes that form granulomatous lesions accompanied by localized infiltration of inflammatory cells [1,2,3]. LCH is currently considered to be an inflammatory neoplastic disease of myeloid origin. The incidence rate of LCH in children aged 0–15 years ranges from 2 to 3 per 500,000 annually [4,5]. LCH can affect nearly every organ in the body, with the number and location of affected organs varying significantly. The bones, skin, and pituitary gland are the most commonly impacted [6,7]. The clinical presentation of LCH is heterogeneous, and there are many pitfalls or difficulties in pathological diagnosis, often leading to misdiagnosis or missed diagnosis [8,9,10]. The involvement of the liver, spleen, or bone marrow is associated with the highest risk of death in children, so the liver, spleen, or bone marrow are classified as high-risk organs [6,11]. A recent study has found that severe hematological involvement (SHI, defined as hemoglobin ≤7 g/dL and platelets ≤20 G/L) in LCH is associated with distinct clinical features, higher complication rates, and poorer outcomes [12]. The progression of LCH is primarily driven by aberrant activation of the MAPK signaling pathway, with the BRAF^V600E^ mutation being particularly significant, detected in approximately 38% to 68% of pediatric patients [7,13,14]. The study revealed that MAPK/PI3K pathway alterations were present in 77.6% of adult LCH patients [15].

There are three rapidly accelerating fibrosarcoma (RAF) in mammalian cell genes, namely ARAF, BRAF, and CRAF [3,16]. BRAF can be activated by the oncogene RAS and drives cell proliferation via the RAS-RAF-MEK-ERK pathway, which is activated in all LCH patients. The BRAF^V600E^ mutations result in sustained activation of BRAF, leading to tumorigenesis by promoting cellular differentiation, resistance to apoptosis, and oncogene-induced senescence [7,17]. Tumorigenesis may also arise from other activating mutations in BRAF, such as the BRAF^V600DLAT^ mutation and the BRAF^V600D^ mutation. MAP2K1, encoding the MEK1 protein, is the second most commonly mutated target after BRAF. The mutual exclusivity of BRAF and MAP2K1 mutations in LCH requires further validation [18,19].

Liquid biopsy uses a minimally invasive approach to obtain samples, such as blood or bone marrow, for testing. Circulating tumor DNA (cfBRAF^V600E^ may become a key indicator for changing) is referred to as a “liquid biopsy” because it detects cancer-related DNA fragments in the bloodstream, offering a minimally invasive alternative to traditional tissue biopsies. This technique holds bright promise for early detection, diagnosis, prognostic analyses, and real-time monitoring of response to treatment and recurrence of cancerous cancers [20,21]. In recent years, numerous studies have highlighted the potential of liquid biopsies for tracking molecular events, evaluating minimal residual disease, and assessing prognosis in LCH [22,23,24]. Circulating free DNA (cfDNA) refers to DNA fragments freely circulating in the bloodstream and originating from any cell type, not exclusively from tumors. As a minimally invasive test, cfDNA promises to be used as an alternative adjunct to currently available modalities. To investigate the correlation between cfBRAF^V600E^ molecular response, radiographic response, and LCH progression, we analyzed 119 plasma and tissue samples. To investigate the correlation between cfBRAF^V600E^ molecular response, radiographic response, and LCH progression, we analyzed 119 plasma and tissue samples. Our paper aims to explore the concordance between circulating tumor DNA (ctDNA) testing and imaging analysis in the monitoring of Langerhans cell histiocytosis (LCH) to more accurately determine treatment outcomes and the disease state in children. In this work, we focus on how these two modalities can complement each other to enhance diagnostic accuracy and patient management, and the final goal is to examine the broader role of ctDNA in monitoring the disease.

## 2. Methods

### 2.1. Study Design

**Figure 1:**
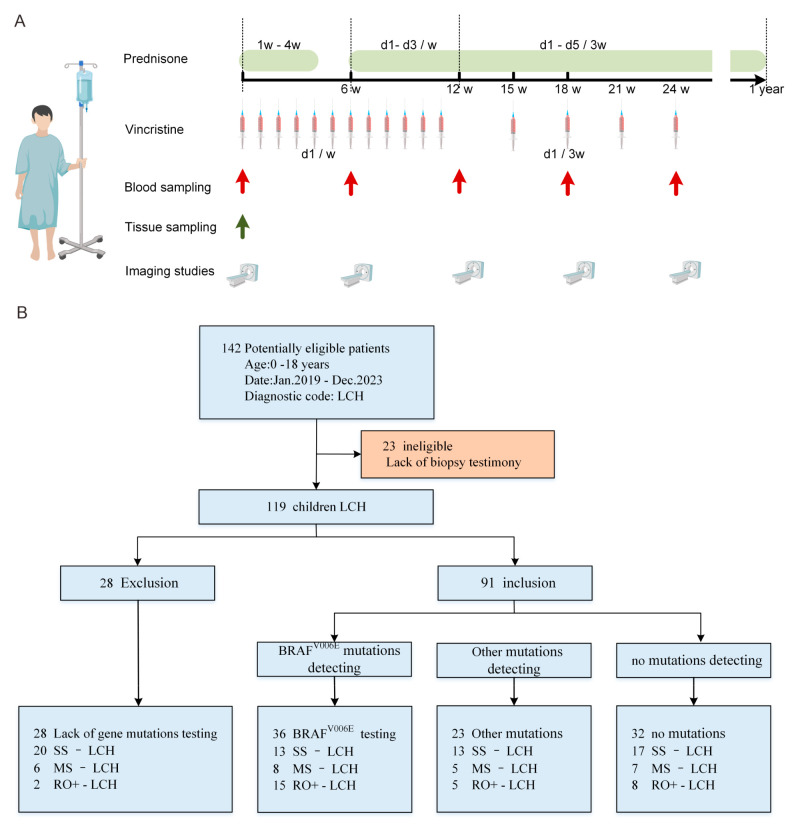
(**A**) The Chinese Children’s Histiocytic Group (CCHG)-Langerhans Cell Histiocytosis (LCH)-2019 treatment regimen. (**B**) Flow diagram of patients: 119 newly diagnosed pediatric LCH cases (1–18 years of age) were studied between 2019 and 2023. SS-LCH: single system-LCH; MS-LCH: multisystem LCH; RO+-LCH: risk organ invasion LCH.

The enrollment criteria included pathologically confirmed LCH and no prior exposure to chemotherapy before the first-line protocol. All children received the Chinese Children’s Histiocytic Group (CCHG)-LCH-2019 treatment regimen, consisting of mainline chemotherapy with vincristine (1.5 mg/m^2^) and prednisone (40 mg/m^2^). This regimen involved 6 weeks of induction therapy across two courses, followed by maintenance therapy with or without 6-Mercaptopurine (50 mg/m^2^) monohydrate for a total duration of one year. At the time of initial diagnosis, ten unstained pathology slides were selected for mutation detection via lesion tissue DNA (ltDNA), and 10 mL of peripheral blood was drawn for cfDNA testing prior to chemotherapy. ltDNA is typically performed post-diagnosis using Next-Generation Sequencing (NGS) or real-time Quantitative Polymerase Chain Reaction (qPCR). cfDNA was collected and reviewed every 6 weeks during the later stage with specific qPCR and droplet digital PCR (ddPCR) (Figure 1A).

Imaging studies include ultrasound (US), CT, MRI, and positron emission tomography-CT (PET-CT) of involved and suspected lesions. The involvement of LCH on imaging findings was categorized based on anatomical location and descriptions from previous studies [4,10]. Lesion tissue that was not measurable by imaging, such as skin lesions, was excluded from the assessment of detectability. A quantitative analysis of lesions based on the US, CT, MRI, and PET-CT data was retrospectively obtained in consensus from 2 radiologists with more than 10 years of experience. A third radiologist with more than 20 years of experience resolved all divergences between the two reviewers. Except for the diagnosis of LCH, the patient’s clinical data and other imaging results were blinded to the radiologists during the first to the last follow-up. The main content of the imaging analysis included the following: (1) density/signal intensity, size, border, morphology, enhancement pattern, and degree of enhancement of the lesion; (2) extent of the lesion, including its location, involvement of peripheral blood vessels, and lymph nodes; (3) evolution of the lesion site, with comparative analyses during the follow-up period; (4) identification of suspicious lesions, determining whether they are tumors or nonspecific abnormalities; (5) measurement of the pituitary gland height; (6) length and thickness of the liver and spleen and assessment of fibrosis, characterized by patchy, nodular, or fused non-enhanced abnormalities diffusely distributed in the liver on non-enhanced images.

The methods used to assess the disease status at the 6th week included routine blood counts, biochemical tests, height measurements, urine osmolality, and endocrine tests (if necessary), along with imaging of affected and suspected areas. Basic clinical information was collected, including age, sex, disease extent at diagnosis, involvement lesions, 3-year progression-free survival (PFS) rate, and the response to chemotherapy at the 6th week.

### 2.2. Study Enrollment and Participants

This study retrospectively screened 142 pediatric LCH cases (1–18 years of age) admitted to X Hospital from 2019 to 2023. Of these, 119 newly diagnosed pediatric LCH cases were consecutively enrolled. Tumor tissue DNA (ltDNA) and cfDNA were prepared from 91 patients. Lab data from 23 patients were excluded for lacking LCH biopsy testimony (Figure 1B). The diagnostic criteria, the clinical classification, and the classification of organs at risk for LCH are detailed in Supplementary Table S1. The study was approved by the Ethics Committee, and informed consent was obtained from the parent or legal guardian of each individual participant included in the study. All patients were assessed for disease status in the 6th, 12th, 26th, and 52nd weeks to inform the subsequent course of the treatment plan. Disease status was classified as follows: (1) no active disease (NAD): complete resolution of symptoms with normal imaging findings; (2) active disease (AD): (i) active disease-better (AD-B): some of the lesions improved, without any worsening of existing lesions; (ii) active disease-intermediate (AD-I): some lesions improved while others worsened, or new lesions appeared; (iii) active disease-stable (AD-S): original symptoms and signs remain without the emergence of new lesions; (iv) active disease-worse (AD-W): worsening of existing symptoms or the appearance of new lesions. Patients presenting with a solitary skin involvement or a single non-central nervous system (CNS) bone involvement will not initially undergo chemotherapy. Instead, they will be evaluated every 3 months and will commence the first-line treatment regimen only if the evaluation outcome indicates AD-I or AD-W. PFS rate was defined as the percentage of patients who did not experience LCH progression, disease relapse, or death. LCH relapse is defined as the infiltration of new lesions after the original LCH lesion has been cured. LCH progression was defined as (a) aggravation or lack of improvement in the affected lesion, (b) partial improvement or worsening of lesions, or the emergence of new lesions, and (c) initiation of a second/further-line chemotherapy [25,26]. We defined the increase in the proportion of BRAF^V600E^ mutations during follow-up as cfDNA molecular progression, and we defined radiologic progression as that patient had radiographic disease progression. cfDNA molecular response, radiologic response, and clinical outcomes were assigned in a blinded manner.

### 2.3. Quantification of cfDNA and ltDNA by PCR or NGS

For next-generation sequencing, the library was prepared by fragmenting nucleic acids through ultrasonic disruption. The target region of Langerhans cell histiocytosis-related genes was identified using a multifactorial algorithm. Target sequences were captured using high-performance liquid chromatography probes. The library then underwent paired-end sequencing on the Illumina sequencing platform. The sequenced sections were compared with the NCBI hg19 reference genome (Supplementary Table S2) using BWA software 0.7.17. Mutation detection was performed using MuTect2 software 4.1.9.0, while gene fusion prediction was conducted with Factera software 1.4.4. For the qPCR assay, nucleic acids were extracted from the samples using an RNA extraction kit. A 15 μL mixture was prepared according to the reverse transcription system, consisting of 10 μL Master MIX, 3 μL probe, and 2 μL cDNA. This mixture was applied to the digital chip and reverse-transcribed into cDNA via PCR. The resulting data were then imported into the analysis software, which calculated the number and proportion of mutations. The total reaction volume for ddPCR analysis was 20 µL, and droplets were amplified using a QX200 droplet generator (Bio-Rad, Hercules, CA, USA) and then read on a two-fluorescence detector (QX200, Bio-Rad). The mutant copy number and mutant allele score of the samples were determined by QuantaSoft version 1.7.4 (Bio-Rad). The mutation rate was defined as the percentage of detection-quantified BRAF^V600E^ mutant fragments compared to the detected wild-type BRAF fragments. The proportion of mutations < 0.001% was defined as negative (−), and the proportion of mutations ≥ 0.001% was defined as positive (+).

### 2.4. Statistical Analysis

Statistical analysis was conducted using the GraphPad Prism version 9 and the SPSS 23.0. All continuous data were evaluated using either a t-test or a non-parametric rank-sum test, as appropriate. The Chi-squared (χ^2^) test was used for the analysis of the enumeration data of two groups. A *p*-value < 0.05 was considered statistically significant. The t-test was applied to compare the means of two sets of data of the continuous variables, expressed as mean ± standard deviation. The other continuous baseline characteristics were compared using a non-parametric rank-sum test and reported as median (interquartile range).

## 3. Results

### 3.1. Baseline Characteristics of Patients

From 2019 to 2023, a total of 119 eligible patients were randomized into the study. The cohort comprised 77 males (64.7%) and 42 females (35.3%), with a median age at diagnosis of 5 years (range: 2–7.1 years). Patients were categorized as 76 (63.8%) SS LCH, 13 (10.9%) MS LCH, and 30 (25.2%) RO+ LCH. The predominantly involved systems included bone (*n* = 100), skin (*n* = 14), liver (*n* = 22), spleen (*n* = 15), central nervous system (*n* = 47), and lymph nodes (*n* = 18). Of the 91 patients who completed cfDNA and ltDNA tests, 35 (38.5%) had the ltBRAF^V600E^ mutation, and 25 (27.5%) had the cfBRAF^V600E^ mutation. Clinical characteristics were compared between the 91 patients who underwent ltDNA and cfDNA testing and the 28 patients who were excluded due to missing detection. No significant differences were observed in clinical characteristics such as age, gender, disease extent at diagnosis, organ involvement lesions, and 3-year PFS rate (Table 1).

### 3.2. Correlation of the Dynamics of cfDNA Molecular Responses with Clinical Outcomes

**Figure 2:**
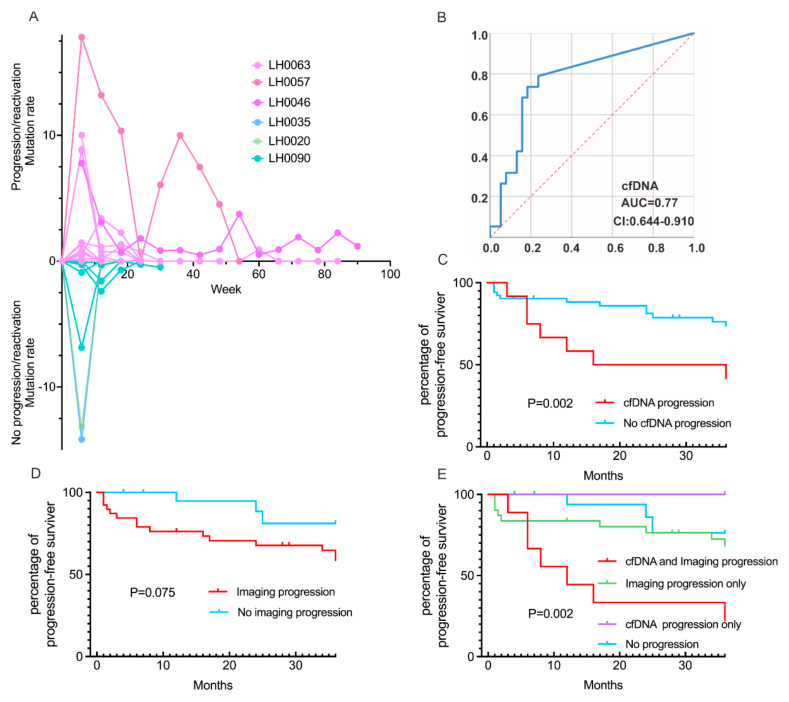
(**A**) Spider plot demonstrating sustained mutation rates in 25 cfBRAF^V600E^-positive children with LCH. Patients were divided into two groups based on whether they experienced disease progression. Patients are represented either above or below the horizontal axis. Each curve represents an individual patient, and each point reflects the mutation value of the patient at a specific time. (**B**) ROC analyses of the truncated value of cfBRAF^V600E^. AUC: Area Under the Curve; CI: Confidence Interval. (**C**–**E**) Patients with either cfBRAF^V600E^ or imaging progression had a significantly shorter progression-free survival (PFS) compared to those without cfBRAF^V600E^ or imaging progression.

In this study, we dynamically monitored cfBRAF^V600E^ status at multiple time points during chemotherapy in children with LCH and evaluated the correlation between cfDNA molecular responses and clinical outcomes. The mutation values of cfBRAF^V600E^ are displayed in a spider plot (Figure 2A). Based on the clinical outcomes, the children were categorized into progression/reactivation and non-progression/reactivation groups. In the progression/reactivation group, LH0057 and LH0046 showed persistent cfBRAF^V600E^ positivity, with LH0057 demonstrating notably higher and recurrent strong positivity. The cfDNA was identified as an independent risk factor for poor prognosis of LCH. Further ROC analysis confirmed cfBRAF^V600E^ as a reliable predictor of PFS, with an Area Under the Curve (AUC) of 0.77 (95% Confidence Interval (CI): 0.64–0.91). The optimal cut-off value for cfBRAF^V600E^ was determined to be 0.03%, with a sensitivity of 0.789 and a 1-specificity of 0.237 (Figure 2B).

### 3.3. Concordance of cfDNA Molecular and Radiographic Responses on Clinical Outcomes

Twelve (48.0%) of twenty-five patients experienced a progression in the proportion of cfBRAF^V600E^ mutations during follow-up, and seven (58.3%) of these children exhibited LCH progression/reactivation, compared to 12 (21.8%) of 55 patients in the no molecular response group who showed LCH progression/reactivation. The progression/reactivation rate was significantly higher in children with cfDNA molecular progression compared to those without (58.3% vs. 21.8%, *p* = 0.011; Table 2). Patients with molecular progression attained a shorter PFS compared to those without (PFS expectancy 22.25 months versus 25.13 months, respectively; Figure 2C). In the imaging progression group, 16 (41%) of 39 children exhibited LCH progression/reactivation, while in the group without imaging progression, only 3 (15%) of 20 children showed LCH progression/reactivation. There was a statistically significant difference in the recurrence rate between the two groups (41% vs. 15%, *p* = 0.045; Table 2). Patients with imaging progression also attained a shorter PFS than those without (PFS expectancy 24.33 months versus 26.98 months, respectively; Figure 2D).

We then compared and analyzed the combined effect of molecular and imaging progression in assessing clinical outcomes in children LCH patients. Among the eight children in the cfBRAF^V600E^ and imaging progression group, six (75%) showed LCH progression/reactivation. In the group with neither molecular nor imaging progression, consisting of 18 cases, only 3 (16.7%) exhibited progression or reactivation. The molecular and imaging progression group was significantly more likely to experience relapse or progression compared to the group without molecular and radiologic progression (75% vs. 16.7%, *p* = 0.004; Table 2). Patients with cfBRAF^V600E^ and imaging progression attained a shorter PFS than those without (PFS expectancy 17.67 months versus 24.67 months, respectively; Figure 2E). The sensitivity of molecular progression in predicting the best overall response (BOR) of radiologic progression was 20.5%, with a specificity of 85.7% and a kappa value of 0.048.

**Figure 3:**
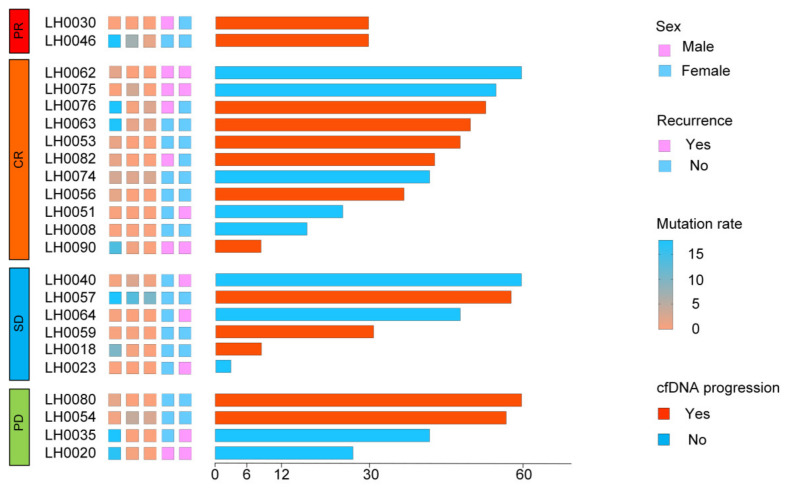
Analyses of PFS by cfDNA molecular response and radiographic response assessment. The swimmer plot depicts the timing of radiographic response assessment, molecular response trajectory, and PFS for each evaluable patient with an increased percentage of cfDNA mutations. Patients were grouped according to their radiographic response and categorized as CR, PR, SD, and PD. The 5-column heatmap represents, from left to right, the proportion of cfDNA mutations, sex, and recurrence at time points C1M1, C2M1, and C3M1, respectively. Bars represent the PFS of the affected children. CR: complete response; PR: partial response; SD: stable disease; PD: progressive disease; C1M1: the 6th-week treatment timepoint; C2M1: the 12th-week treatment timepoint; and C3M1: the 18th-week treatment timepoint.

**Figure 4:**
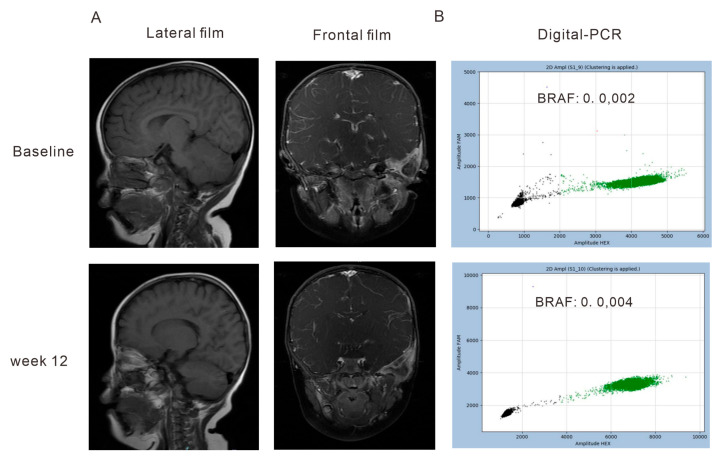
Imaging features of left temporal bone lesions and proportion of mutations detected by digital PCR. A 2-year-old boy with a temporal bone lesion diagnosed as multisystem Langerhans cell histiocytosis–risk organ invasion. (**A**) T1-weighted imaging (T1WI) from lateral and frontal views shows the temporal bone and its surrounding tissues at the start of treatment and after 12 weeks of treatment. (**B**) The BRAF^V600E^ mutation rate of the child detected by digital PCR after 6 and 12 weeks of treatment.

The overall radiographic response was evaluated in the 12th week. Tumor responses were classified into two groups: radiographic response (complete response (CR) and partial response (PR)) and no radiographic response (stable disease (SD) and progressive disease (PD)). The maximal mutant allele fraction (max MAF) was tracked at the 6th-weektreatment timepoint (C1M1) to on-therapy 12th- and 18th-week timepoints (C2M1 and C3M1). Among the patients, LH0057, LH0035, and LH0020 exhibited high levels of cfBRAF^V600E^ mutations and showed no radiographic response (SD and PD), with LH0057 demonstrating persistent cfBRAF^V600E^ positivity across all time points (Figure 3). Imaging analysis showed a stable left temporal bone lesion in LH0008 (Figure 4A), and digital PCR results revealed an increasing proportion of mutations (Figure 4B). In contrast, patients with radiographic responses (CR and PR) showed predominantly low cfBRAF^V600E^ mutation rates, as seen in the heatmap (Figure 3).

### 3.4. Comparison of cfBRAF^V600E^ and ltBRAF^V600E^ Status in Relation to LCH Clinical Presentations and Outcomes

A total of 25 cases of cfBRAF^V600E^ and 35 cases of ltBRAF^V600E^ tested positive in the study. Among them, 24 cases were positive for cfBRAF^V600E^ and ltBRAF^V600E^. Eleven cases were positive for ltBRAF^V600E^ but negative for cfBRAF^V600E^. This discrepancy may stem from the differing biological sources for ltDNA and cfDNA. While ltDNA detects genetic mutations directly within tumor tissues, cfDNA identifies free tumor DNA in peripheral circulating plasma. In cases with a limited number of tumor cells, cfDNA may yield false-negative results, necessitating multiple tests or a combination of imaging and other modalities for accurate tumor detection. One patient was positive for cfBRAF^V600E^ at diagnosis but negative for ltBRAF^V600E^. Additionally, 33 children had undetectable mutations during follow-up.

**Figure 5:**
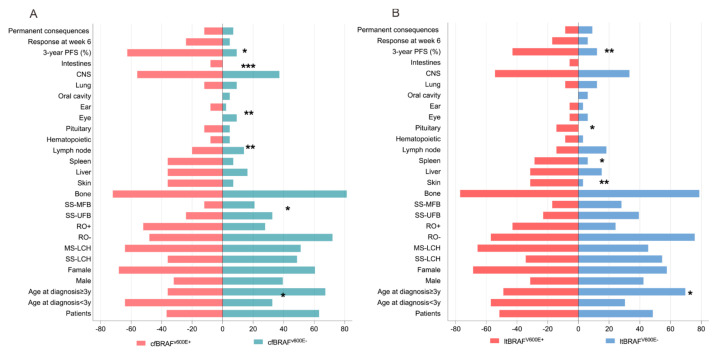
(**A**) Clinical characteristics, treatment response, and outcomes between cfBRAF^V600E^-negative and cfBRAF^V600E^-positive. (**B**) Clinical characteristics, treatment response, and outcomes between ltBRAF^V600E^-negative and ltBRAF^V600E^-positive. * *p* < 0.05, ** *p* < 0.01, *** *p* < 0.001. Y: Year; SS-LCH: single system Langerhans cell histiocytosis; MS-LCH: multisystem Langerhans cell histiocytosis; RO-: no risk organ invasion; RO+: risk organ invasion; CNS: central nervous system; PFS: progression-free survival; SS-UFB: unifocal bone disease; SS-MFB: multifocal bone disease.

**Figure 6:**
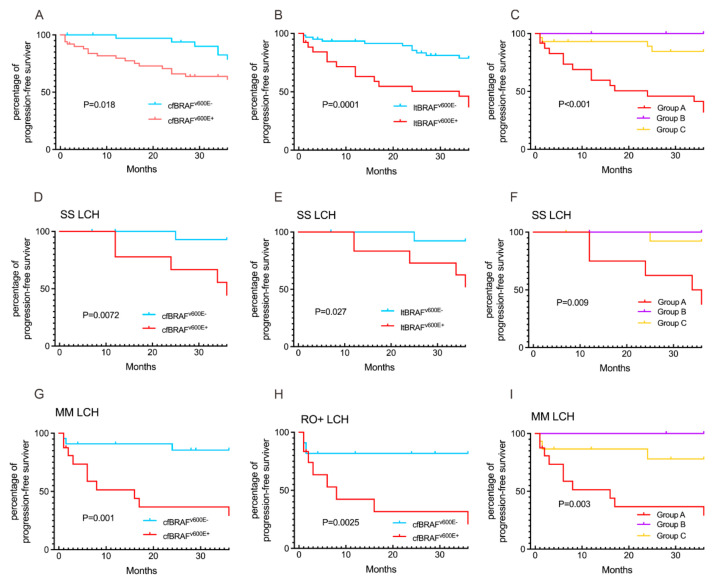
(**A**) Three-year progression-free survival (PFS) in the cfBRAF mutation cohort of 68 patients. (**B**) Three-year PFS in the cfBRAF mutation cohort of 68 patients. (**C**) Three-year PFS in the BRAF mutation cohort of 68 patients. (**D**,**G**,**H**) Patients with cfBRAF^V600E^-positive had a longer PFS compared to patients with cfBRAF^V600E^-negative in subtypes SS-LCH and MS-LCH, RO+ LCH. (**F**,**I**) Three-year PFS in the BRAF mutation cohort of 68 patients in subtypes SS-LCH and MS-LCH. (**E**) Patients with ltBRAF^V600E^-positive had a longer PFS compared to patients with ltBRAF^V600E^-negative in subtypes SS-LCH. SS-LCH: single system Langerhans cell histiocytosis; MS-LCH: multisystem Langerhans cell histiocytosis; RO+-LCH: risk organ invasion Langerhans cell histiocytosis. Group A: positive for both ltBRAF^V600E^ and cfBRAF^V600E^; Group B: positive for either ltBRAF^V600E^ or cfBRAF^V600E^; and Group C: negative for both mutations.

The cfBRAF^V600E^ mutation was significantly associated with younger age at diagnosis (median 2.17 years [1.5–7] vs. 5.08 years [2,3,4,5,6,7,8,9], *p* = 0.036), a higher proportion of patients under 3 years old (64% vs. 32%, *p* = 0.012), a greater proportion of RO+ LCH (52% vs. 27.9%, *p* = 0.047), involvement of the skin (36% vs. 7%, *p* = 0.002) and spleen (36% vs. 7%, *p* = 0.002), the treatment response at the sixth week (24% vs. 4.7%, *p* = 0.002), and a higher 3-year PFS rate (37.5% vs. 90.7%, *p* < 0.0001) compared to patients without the mutation (Table 3, Figure 5A). Similarly, the ltBRAF^V600E^ was significantly associated with a higher proportion of patients under 3 years old (57.1% vs. 30.3%, *p* = 0.026), the involvement of the skin (31.4% vs. 3%, *p* = 0.002) and spleen (28.6% vs. 6.1%, *p* = 0.015), and pituitary (14.3% vs. 0.0%, *p* = 0.024), as well as a better 3-year PFS rate (57.1% vs. 87.9%, *p* = 0.005) compared to patients without the mutation (Table 3, Figure 5B). Additionally, we observed significant differences among patients in Group A (positive for both ltBRAF^V600E^ and cfBRAF^V600E^), Group B (positive for either ltBRAF^V600E^ or cfBRAF^V600E^), and Group C (negative for both mutations) regarding the proportion of children under 3 years of age (66.7%, 33.3%, and 31.3%, *p* = 0.021), skin involvement (37.5%, 16.7%, and 3.1%, *p* = 0.003), and spleen involvement (37.5%, 8.3%, and 6.3%, *p* = 0.005), and the 3-year PFS rate (34.8%, 100%, and 87.5%, *p* < 0.001) (Table 3). The cfBRAF^V600E^ was related to 3-year PFS in patients with SS-LCH, MM-LCH, and RO+ LCH (Figure 6A,D,G,H), while the ltBRAF^V600E^ was related to 3-year PFS in patients with SS-LCH (Figure 6B,E). There were significant differences among Groups A, B, and C in patients with SS-LCH and MM-LCH (Figure 6C,F,I).

### 3.5. MAP2K1 and BRAF^exon12^ Mutations in Relation to LCH Clinical Presentations and Outcomes

**Figure 7:**
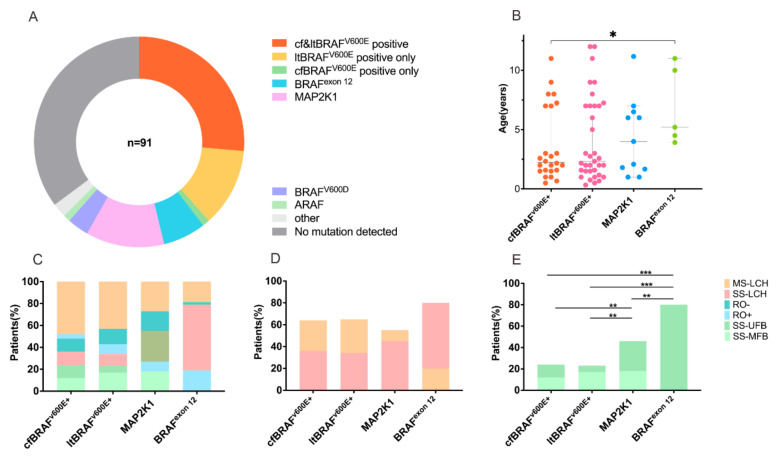
Clinical characteristics in LCH children with cfBRAF^V600E^, ltBRAF^V600E^, BRAF^exon12^, or MAP2K1 mutations. (**A**) The pie chart shows the mutational status of the 91 patients in our cohort. (**B**) Dot plot shows age at diagnosis of patients with cfBRAF^V600E^, ltBRAF^V600E^, BRAF^exon12^, or MAP2K1 mutations. Error bars depict medians with interquartile ranges. (**C**–**E**) Bar charts demonstrate the percentage of patients with cfBRAF^V600E^, ltBRAF^V600E^, BRAF^exon12^, or MAP2K1 mutations having subtypes at LCH diagnosis. SS-LCH: single system Langerhans cell histiocytosis; MS-LCH: multisystem Langerhans cell histiocytosis; RO-: no risk organ invasion; RO+: risk organ invasion; SS-UFB: unifocal bone disease; SS-MFB: multifocal bone disease. * *p* < 0.05, ** *p* < 0.01, *** *p* < 0.001.

Among the 119 patients enrolled, 25 cases of cfBRAF^V600E^ and 35 cases of ltBRAF^V600E^ were positive. ltMAP2K1+ mutations were found in 11 (19.6%) patients, ltBRAF-exon12+ in 6 (10.7%) patients, ltBRAF-V600D+ in 3 patients, and 1 patient had an ARAF mutation; moreover, 33 patients with no detected mutations served as the control group (Figure 7A). The clinical characteristics, treatment response, and outcome of MAP2K1 and BRAF^exon12^ mutations are specifically analyzed and discussed, which serves as a reference for patients with detectable mutations in the MAPK signaling pathway other than BRAF^V600E^. Compared with children without mutations, patients with MAP2K1 mutations had significantly more ear involvement (27.3% vs. 1%; *p* = 0.045) (Supplementary Table S3). No significant differences in clinical characteristics, treatment response, and outcomes were observed between the BRAF^exon12^ mutations group and the no mutation group (Supplementary Table S3). The consistency between the clinical characteristics of different mutations in cfDNA and ltDNA assays was compared. Regarding this, cfBRAF^V600E^ mutations appeared to correlate with younger age at diagnosis compared to ltBRAF^V600E^, MAP2K1, or BRAF^exon12^ mutations (3.55 ± 3.11, 4.10 ± 3.54, 4.38 ± 3.24 vs. 6.92 ± 3.24 years, respectively) (Figure 7B). Figure 7C highlights the proportion of subtype extents in patients with cfBRAF^V600E^, ltBRAF^V600E^, BRAF^exon12^, or MAP2K1 mutations. Children with BRAF^exon12^ mutations were more likely to have SS-LCH (83.3% vs. 36%, 34.3%, 54.5%) and less likely to have MS-LCH (16.7% vs. 64%, 65.7%, 45.5%) compared to patients with cfBRAF^V600E^, ltBRAF^V600E^, or MAP2K1 mutations (Figure 7D). Regarding bone involvement subtypes, patients with BRAF^exon12^ mutations had the highest prevalence of SS-UFB (83.3% vs. 24%, 22.9%, 54.5%; *p* < 0.05) and the lowest prevalence of SS-MFB (0% vs. 12%, 17.1%, 18.2%) compared to those with cfBRAF^V600E^, ltBRAF^V600E^, or MAP2K1 mutations (Figure 7E).

## 4. Discussion

A critical challenge in optimal tumor monitoring lies in the heterogeneity of tumor clinical presentations, the limitations of imaging in detecting measurable residual disease, and the difficulty of real-time assessment to guide treatment regimens [1,6,22,27]. Liquid biopsy represents a significant advancement, shifting from the surgical removal of tumor tissue specimens to the less invasive extraction of blood samples by nursing staff. This marks a major step forward in tumor detection. Liquid biopsy has made tumor load monitoring both rapid and easy to perform in real time [4,28,29].

Including the significance of cfDNA molecular responses, substantial progress has been made in the application of cfDNA in LCH [23,30,31]. Nonetheless, more information still needs to be duly confirmed. Our paper aims to explore the concordance between cfDNA and imaging analysis in the monitoring of LCH. The combination of molecular and radiographic responses proved to be more informative in predicting PFS. We found that children with LCH who exhibited both cfBRAF^V600E^ progression and imaging progression had significantly lower PFS compared to those without, as well as those with only cfBRAF^V600E^ progression or only imaging progression. Both cfBRAF^V600E^ progression and imaging progression can be used as markers of poor prognosis or the need for intensified treatment in children with LCH. ctDNA captures tumor DNA from peripheral circulation and offers superior sensitivity and specificity for detecting microscopic residual disease compared to imaging analysis. It enables real-time monitoring of tumor dynamics, facilitating earlier detection of recurrence. Additionally, ctDNA testing eliminates the need for invasive procedures and minimizes radiation exposure associated with imaging, thereby enhancing patient comfort and safety.

This paper is based on data visualization and a survival analysis model analyzing long-term ctDNA and imaging data of 119 children with LCH. This resulted in 25 cases of cfBRAF^V600E^ positivity, 35 cases of ltBRAF^V600E^ positivity, 21 cases with other gene mutations, and 33 cases with no detectable mutations; these totals served as informative information to obtain the concordance between cfBRAF^V600E^ mutations and imaging responses, as well as the relationship between cfBRAF^V600E^ and ltBRAF^V600E^ status in relation to LCH clinical presentation and prognosis. Compared to parents included in the Toronto cohort, we observed a higher frequency of positive ltBRAF^V600E^ (38.5% vs. 31.53%). Similarly, our cohort demonstrated a higher frequency of detectable cfBRAF^V600E^ mutations compared to the French cohort (27.5% vs. 10.5%) [23,31]. These differences are likely related to ethnic and geographical factors. In this study, we also aimed to use cfDNA-detected mutations in LCH as an enrichment strategy to identify individuals at high risk for disease progression, recurrence, response to chemotherapy, and early detection of organ-at-risk involvement, enabling timely preventive measures [28]. We found that cfDNA progression was closely related to relapse and progression. We propose that cfBRAF^V600E^ progression can serve as an indicator for treatment escalation in LCH. A prospective study could be conducted to adjust treatment regimens early in children with cfDNA progression in LCH. In addition, cfBRAF^V600E^ has been reported as an independent risk factor for LCH in adults and children, and we determined the cut-off value for cfBRAF^V600E^ in this context.

The cfBRAF^V600E^ and ltBRAF^V600E^ mutations were associated with a higher proportion of children under 3 years of age, skin and spleen involvement, and a lower 3-year PFS rate. In addition, we observed 11 cases that tested positive for ltBRAF^V600E^ but negative for cfBRAF^V600E^. This discrepancy is attributed to the differing bio-materials of ltDNA and cfDNA. While ltDNA directly detects mutations within tumor tissues, cfDNA identifies free tumor DNA circulating in peripheral plasma. These factors contribute to a higher incidence of false-negative results with cfDNA when tumor cell numbers are limited. However, these same characteristics position cfDNA as a more promising indicator of prognosis and treatment efficacy [32]. This observation was further validated in our study. We found that cfBRAF^V600E^-positive children were diagnosed at a younger age and had more RO+ involvement. These children also had a poorer treatment response at the sixth week and were more likely to require a change in regimen, whereas a distinction was not observed with ltBRAF^V600E^. Additionally, cfBRAF^V600E^-positive children had a lower 3-year PFS in both RO+-LCH and MS-LCH subtypes, a unique finding that distinguishes ltBRAF^V600E^-positive patients. No significant differences were found in clinical classification, tumor invasion sites, response to treatment, clinical outcomes, or permanent damage between children with MAP2K1 or cfBRAF^exon12^ mutations and those without detectable mutations.

In the future, at the time of initial diagnosis, cfDNA will be the preferred monitoring method in the treatment regimen in LCH clinical guidelines for small or undersized lesions. As our findings showed, in RO+-LCH and MS-LCH subtypes, the 3-year-PFS was significantly lower in the cfBRAF^V600E^-positive group than in the cfBRAF^V600E^-negative group. We demonstrated that cfBRAF^V600E^ predicted treatment response, whereas ltBRAF^V600E^ did not. Our study underscored the importance of cfDNA molecular responses in childhood LCH, and we successfully established a cut-off value for cfDNA molecular response. cfDNA holds potential for routine clinical application across various tumors. For instance, children with leukemia face high risks of morbidity and mortality from pancreatitis during treatment; early detection of pancreatic DNA in their peripheral blood could enable timely intervention, helping to prevent the onset of pancreatitis. This study has several limitations. First, the number of BRAF-positive patients decreased following the final detailed categorization. To better understand the potential impact of MAPK pathway mutations, it is necessary to gather a larger sample size and cohort of LCH patients for further validation. Additionally, as our center is located in the central region of the country, data from multiple centers should be included in future studies to mitigate potential geographical biases. Although the consistency of ctDNA testing and imaging analysis for LCH surveillance was initially evaluated in this paper, since ctDNA testing can sometimes yield false negatives, extending the follow-up period in future studies is crucial to reduce chance bias and ensure more robust results, which will potentially increase the number of investigators involved.

## 5. Conclusions

We revealed that the concordance between cfDNA molecular response and radiographic progression was highly relevant for tracking pediatric LCH progression and recurrence. The group with both molecular and imaging progression had a significantly higher likelihood of relapse or disease progression compared to the group without progression (*p* < 0.05). PFS was notably shorter in patients exhibiting both radiologic and cfDNA molecular progression (17.67 vs. 24.67 months, *p* < 0.05) compared to those without. Our study highlighted the significance of the molecular response of cfBRAF^V600E^, as we found that in RO+-LCH and MS-LCH subtypes, the 3-year PFS was significantly lower in the cfBRAF^V600E^-positive group compared to the cfBRAF^V600E^-negative group, a distinction not observed with ltBRAF^V600E^. Furthermore, the cut-off value of cfDNA molecular response is established. Our findings support the implementation of liquid biopsy in children with LCH and further advance the roadmap of evidence, paving the way for cfDNA molecular responses to play an increasing role in clinical decision-making for these patients.

## Figures and Tables

**Table 1:** Clinical characteristics in 119 children included or excluded in this study.

	ltDNA and cfDNA	Missing Detection	*p*
Patients, *n*	91	28	
Age at diagnosis, *y*	4.0 (2–7)	5.37 (3.25–9.25)	0.098
Gender, *n* (%)			
Male	59 (64.8)	20 (71.4)	0.518
Female	32 (35.2)	8 (28.6)	
Disease extent at diagnosis, *n* (%)			
SS-LCH	56 (61.5)	20 (71.4)	0.174
MS-LCH-RO-	7 (7.7)	4 (14.3)	
MS-LCH-RO+	28 (30.8)	4 (14.3)	
Involvement, *n* (%)			
Bone	75 (82.4)	25 (89.3)	0.386
Skin	12 (13.2)	2 (7.1)	0.385
Liver	20 (22.0)	2 (7.1)	0.077
Spleen	14 (15.4)	1 (3.6)	0.100
Lymph node	16(17.6)	2 (7.1)	0.178
Hematopoietic	6 (6.6)	0 (0.0)	0.163
Pituitary	6 (6.6)	1 (3.6)	0.552
Eye	4 (4.4)	3 (10.7)	0.214
Ear	6 (6.6)	1 (3.6)	0.552
Oral cavity	3 (3.3)	0 (0.0)	0.330
Lung	9 (9.9)	2 (7.1)	0.661
CNS	40 (44.0)	7 (25.0)	0.073
Intestines	2 (2.2)	1 (3.6)	0.685
3-year PFS rate, %	71.4	89.3	0.054

ltDNA: lesion tissue DNA; cfDNA: cell-free DNA; SS-LCH: single system Langerhans cell histiocytosis; MS-LCH-RO-: multisystem LCH without risk organ invasion; MS-LCH-RO+: multisystem LCH with risk organ invasion; CNS: central nervous system; PFS: progression-free survival.

**Table 2:** Concordance between radiographic and cfDNA molecular response for patients.

	Progression/Reactivation	CR/PR	*p*
Molecular progression, *n* (%)	7 (58.3)	5 (41.7)	0.011 *
No molecular progression, *n* (%)	12 (21.8)	43 (78.2)	
Radiologic progression, *n* (%)	16 (41.0)	23 (59.0)	0.045 *
No radiologic progression, *n* (%)	3 (15.0)	17 (85.0)	
Molecular and radiologic progression, *n* (%)	6 (75.0)	2 (25.0)	0.004 *
No molecular and radiologic progression, *n* (%)	3 (16.7)	15 (83.3)	

CR: complete response; PR: partial response. * *p* < 0.05. A patient who is in active disease–stable (AD-S) at the Week 6 assessment will enter second-line therapy, and the clinical outcome is defined as disease progression. So, progressive/reactivation in the no radiologic progression group was defined as a patient with no increase or decrease in lesions after the first 6 weeks of treatment.

**Table 3:** Correlation of cfBRAF^V600E^ and ltBRAF^V600E^ with clinical characteristics, treatment response, and outcomes.

	cfBRAF^V600E+^	cfBRAF^V600E-^	*p*	ltBRAF^V600E+^	ltBRAF^V600E-^	*p*	Group A	Group B	Group C	*p*
Patients, *n.*	25	43		35	33		24	12	32	
Age at diagnosis, *y*	2.17 (1.5–7)	5.08 (2–9)	0.036 *	2.33 (1.5–7)	5.08 (2–9)	0.068	2.083 (1.5–6)	6.5 (1.38–8.75)	5.04 (2–9)	0.062
<3*y*	16 (64.0)	14 (32.6)	0.012 *	20 (57.1)	10 (30.3)	0.026 *	16 (66.7)	4 (33.3)	10 (31.3)	0.021 *^,b^
≥3*y*	9 (36.0)	29 (67.4)		15 (42.9)	23 (69.7)		8 (33.3)	8 (66.7)	22 (68.8)	
Gender, *n* (%)										
Male	8 (32.0)	17 (39.5)	0.534	11 (31.4)	14 (42.4)	0.347	8 (33.3)	3 (25.0)	14 (43.8)	0.471
Female	17 (68.0)	26 (60.5)		24 (68.6)	19 (57.6)		16 (66.7)	9 (75.0)	18 (56.3)	
Disease extent at diagnosis, *n* (%)										
SS-LCH	9 (36.0)	21 (48.8)	0.304	12 (34.3)	18 (54.5)	0.093	8 (33.3)	5 (41.7)	17 (53.1)	0.330
MS-LCH	16 (64.0)	22 (51.2)		23 (65.7)	15 (45.5)		16 (66.7)	7 (58.3)	15 (46.9)	
Detailed subtype, *n* (%)										
RO-	12 (48.0)	31 (72.1)	0.047 *	20 (57.1)	25 (75.8)	0.105	12 (50.0)	9 (75.0)	24 (75.0)	0.114
RO+	13 (52.0)	12 (27.9)		15 (42.9)	8 (24.2)		12 (50.0)	3 (25.0)	8 (25.0)	
Detailed subtype, *n* (%)										
SS-UFB	6 (24.0)	14 (32.6)	0.455	8 (22.9)	13 (39.4)	0.140	5 (20.8)	3 (25.0)	12 (37.5)	0.373
SS-MFB	3 (12.0)	9 (20.9)	0.352	6 (17.1)	9 (28.1)	0.281	3 (12.5)	2 (16.7)	7 (21.9)	0.707
Involvement										
Bone	18 (72.0)	35 (81.4)	0.368	27 (77.1)	26 (78.8)	0.870	17 (70.8)	11 (91.7)	25 (78.1)	0.364
Skin	9 (36.0)	3 (7.0)	0.002 *	11 (31.4)	1 (3.0)	0.002 *	9 (37.5)	2 (16.7)	1 (3.1)	0.003 * ^b^
Liver	9 (36.0)	7 (16.3)	0.065	11 (31.4)	5 (15.2)	0.114	9 (37.5)	2 (16.7)	5 (15.6)	0.133
Spleen	9 (36.0)	3 (7.0)	0.002 *	10 (28.6)	2 (6.1)	0.015 *	9 (37.5)	1 (8.3)	2 (6.3)	0.005 *^b^
Lymph node	5 (20.0)	6 (14.0)	0.514	5 (14.3)	6 (18.2)	0.663	5 (20.8)	0 (0.0)	6 (18.8)	0.281
Hematopoietic	2 (8.0)	2 (4.7)	0.571	3 (8.6)	1 (3.0)	0.332	2 (8.3)	1 (8.3)	1 (3.1)	0.510
Pituitary	3 (12.0)	2 (4.7)	0.263	5 (14.3)	0 (0.0)	0.024 *	3 (12.5)	2 (16.7)	0 (0.0)	0.048
Eye	0 (0.0)	4 (9.3)	0.116	2 (5.7)	2 (6.1)	0.952	0 (0.0)	2 (16.7)	2 (6.3)	0.121
Ear	2 (8.0)	1 (2.3)	0.272	2 (5.7)	1 (3.0)	0.590	2 (8.3)	0 (0.0)	1 (3.1)	0.579
Oral cavity	0 (0.0)	2 (4.7)	0.274	0 (0.0)	2 (6.1)	0.139	0 (0.0)	0 (0.0)	2 (6.3)	0.663
Lung	3 (12.0)	4 (9.3)	0.724	3 (8.6)	4 (12.1)	0.630	3 (12.5)	0 (0.0)	4 (12.5)	0.660
CNS	14 (56.0)	16 (37.2)	0.132	19 (54.3)	11 (33.3)	0.083	13 (54.2)	7 (58.3)	10 (31.3)	0.128
Intestines	2 (8.0)	0 (0.0)	0.060	2 (5.7)	0 (0.0)	0.163	2 (8.3)	0 (0.0)	0 (0.0)	0.150
3-year PFS rate, *n* (%)	37.5	90.7	<0.001 *	57.1	87.9	0.005 *	34.8	100	87.5	<0.001 *^b^
Response at the 6th week	6 (24.0)	2 (4.7)	0.042 *	6 (17.1)	2 (6.1)	0.296	6 (25.0)	0 (0.0)	2 (6.3)	0.110
Permanent consequences developed during follow-up, *n* (%)	3 (12.0)	3 (7.0)	0.481	3 (8.6)	3 (9.1)	0.940	2 (8.3)	2 (16.7)	2 (6.3)	0.463

Group A: positive for both ltBRAF^V600E^ and cfBRAF^V600E^; Group B: positive for either ltBRAF^V600E^ or cfBRAF^V600E^; and Group C: negative for both mutations. Y: Year; SS-LCH: single system Langerhans cell histiocytosis; MS-LCH: multisystem Langerhans cell histiocytosis; RO-: no risk organ invasion; RO+: risk organ invasion; CNS: central nervous system; PFS: progression-free survival; SS-UFB: unifocal bone disease; SS-MFB: multifocal bone disease.*: *p* < 0.05. ^b^: Bonferroni’s correction was performed. Bold: The difference was statistically significant.

## Data Availability

The datasets generated or analyzed during the study are available from the corresponding author on reasonable request.

## Supplementary Materials

The following supporting information can be downloaded at: https://www.mdpi.com/article/10.3390/children11121449/s1, Supplementary Table S1. Diagnostic criteria, clinical classification, classification of organs at risk. Supplementary Table S2. The NCBI hg19 reference genome. Supplementary Table S3. Correlation of MAP2K1 and BRAF^exon12^ with clinical characteristics, treatment response, and outcome.
