# Supplementary material for: Circulating Tumor DNA Combining with Imaging Analysis for Lesion Detection of Langerhans Cell Histiocytosis in Children
Source: Children (Basel). 2024 Nov 27;11(12):1449. doi: 10.3390/children11121449 (PMC11675047; doi:10.3390/children11121449)
Supplement: Supplementary file 1 [file children-11-01449-s001.zip › children-3295106-supplementary.pdf]

Supplementary Table S1. Diagnostic criteria, clinical classification, classification of organs at risk.

1) Diagnostic criteria
Immunohistochemical staining of tissue in the lesion CD1a positive or CD207 (Langerin) positive
2) Single system-LCH (SS-LCH)
Involvement of only one organ or system (single or multiple lesion) without risk organs
3) Multisystem-LCH (MS-LCH)
Involvement of greater than or equal to 2 organs or systems,
4) Risk organ invasion (RO+)
a) Liver
≥ 3 cm from the lower edge of the ribs on the right midclavicular line, with/without dysfunction
b) Spleen
≥ 2 cm from the lower edge of the ribs on the left midclavicular line
c) hematologic system
i. Haemoglobin < 100 g/L
ii. white blood cell count < 4.0×10^9/L
iii. Platelet count < 100×10^9/L
5) Definition of central nervous system risk parts (CNS)
Craniofacial, orbital or ocular infiltration, or ear involvement

Supplementary Table S2. The NCBI hg19 reference genome.

AKT1	GRB2	MAP2K5	MAP3K3	MAPK14	NRAS	SOS2	CLIP2
MAP2K6	MAP3K4	MAPK3	NTRK1	TAB1	KIF5B	BRAF	IKBKB
MAPK7	PAK1	TAB2	LMNA	CDC42	IL1A	MAP3K1	MAP3K6
TAOK1	ALK	MAP3K11	MAP3K7	MAPK9	KRAS	PIK3CA	RALGDS
CRK	TAOK2	PDGFRA	MAP3K12	MAP3K8	TGFB1	ERBB2	HRAS
KIT	NTRF1	EGFR	MAPKAPK2	MAP2K2	MAP2K4	ARAF	MAP3K5
MAP2K1	MAP4K1	MAP3K13	TNF FGFR1	MAP3K14	MAP4K4	MAP2K7	PAK2
MET	RAF1	TGFBR1	ERBB3	MAP3K20	NFKB1	MAPK8	MAP2K3
MAP3K2	MAPK1	NFKB2	RASA1	TP53	FGFR2	MAPK10	NLK
SOS1	RNF11						

Supplementary Table S3 Correlation of MAP2K1 and $BRAF^{exon12}$ with clinical characteristics, treatment response, and outcome.

	MAP2K1	Group C	*p*	$BRAF^{exon12}$	Group C	*p*
Patients, *n*	11	32		6	32	
Age at diagnosis, *y*	4.38±3.24	5.55±3.64	.354	6.92±3.31	5.61±3.61	.450
<3y	5 (54.5)	10 (31.3)	.473	0 (0.0)	10 (31.2)	.136
≥3y	6 (45.5)	22 (68.8)		6 (100)	22 (68.8)	
Gender, *n* (%)						
Male	4 (36.4)	14 (43.7)	.736	2 (33.3)	14 (43.7)	.489
Famale	7 (63.6)	18 (56.3)		4 (66.7)	18 (56.3)	
Disease extent at diagnosis, *n* (%)						
SS-LCH	5 (54.5)	17 (53.1)	.661	5 (83.3)	17 (53.1)	.180

MS-LCH	6 (45.5)	15 (46.9)		1 (16.7)	15 (46.9)	
Detailed subtype, *n* (%)						
RO-	8 (72.7)	24 (75.0)	.586	5 (83.3)	24 (75.0)	.559
RO+	3 (27.3)	8 (25.0)		1 (16.7)	8 (25.0)	
Detailed subtype, *n* (%)						
SS- UFB	5 (54.5)	25 (78.1)	.728	5 (83.3)	12 (37.5)	.052
SS-MFB	2 (18.2)	7 (21.9)	.583	0 (0.0)	7 (21.2)	.267
Involvement						
Bone	10 (90.9)	35 (81.4)	.656	6 (100)	25 (78.1)	.267
Skin	0 (0.0)	1 (3.1)	.744	0 (0.0)	1 (3.1)	.842
Liver	3 (27.3)	5 (15.6)	.401	1 (16.7)	5 (15.6)	.672
Spleen	1 (9.1)	2 (6.3)	.598	1 (16.7)	2 (6.3)	.412
Lymph node	2 (18.2)	6 (18.8)	.672	0 (0.0)	6 (18.8)	.328
Hematopoietic	1 (9.1)	1 (3.1)	.451	0 (0.0)	1 (3.1)	.842
Eye	0 (0.0)	2 (6.3)	.549	0 (0.0)	2 (6.3)	.706
Ear	3 (27.3)	1 (3.1)	**.045***	0 (0.0)	1 (3.1)	.842
Oral cavity	1 (9.1)	2 (6.3)	.598	0 (0.0)	2 (6.3)	.706
Lung	2 (18.2)	4 (12.5)	.637	0 (0.0)	4 (12.5)	.487
CNS	7 (63.6)	10 (31.3)	.080	2 (33.3)	10 (31.3)	.631
3-year PFS rate, %	63.6	87.5	.172	80.0	87.5	.599
Response at the 6th week	0 (0.0)	2 (6.3)	.549	0 (0.0)	2 (6.3)	.706
Permanent consequences	1 (9.1)	2 (6.3)	.598	0 (0.0)	2 (6.3)	.706

***** *P* < 0.05. Group C: no mutation detected. Y: Year. SS-LCH: Single system-Langerhans Cell Histiocytosis, MS-LCH: Multisystem- Langer-hans Cell Histiocytosis, RO-: No risk organ invasion, RO+: Risk organ invasion. CNS: Central nervous system. PFS: Progression-free survival. SS-UFB: unifocal bone disease, SS-MFB: multifo-cal bone disease.
